# Systematic review of well-being interventions for minority healthcare workers

**DOI:** 10.3389/fmed.2025.1531090

**Published:** 2025-02-21

**Authors:** Tanvi Bafna, Mansoor Malik, Mohua C. Choudhury, William Hu, Christine M. Weston, Kristina R. Weeks, Cheryl Connors, M. Haroon Burhanullah, George Everly, Henry J. Michtalik, Albert W. Wu

**Affiliations:** ^1^Department of Health Policy and Management, Johns Hopkins Bloomberg School of Public Health, Baltimore, MD, United States; ^2^Department of Psychiatry, Johns Hopkins University School of Medicine, Baltimore, MD, United States; ^3^Department of Anesthesiology and Critical Care Medicine, Johns Hopkins School of Medicine, Baltimore, MD, United States; ^4^Johns Hopkins School of Medicine, The Armstrong Institute for Patient Safety and Quality, Baltimore, MD, United States; ^5^Department of Medicine, Johns Hopkins School of Medicine, Baltimore, MD, United States

**Keywords:** well-being, minority healthcare workers, burnout, supportive interventions, peer support

## Abstract

**Introduction:**

Healthcare workers’ well-being is of utmost importance given persistent high rates of burnout, which also affects quality of care. Minority healthcare workers (MHCW) face unique challenges including structural racism and discrimination. There is limited data on interventions addressing the psychological well-being of MHCW. Thus, this systematic review aims to identify interventions specifically designed to support MHCW well-being, and to compare measures of well-being between minority and non-minority healthcare workers.

**Methods:**

We searched multiple electronic databases. Two independent reviewers conducted literature screening and extraction. The Mixed Methods Assessment Tool (MMAT) or Joanna Briggs Institute (JBI) criteria were utilized to assess the methodological quality of studies, based on the study design. Total scores as percentages of criteria met were used to determine overall quality as low (<40%), moderate (40-80%), or high (>80%). For conflicts, consensus was reached through discussion. Meta-analysis was not possible due to heterogeneity of study designs.

**Results:**

A total of 3,816 records were screened and 43 were included in the review. The majority of included studies (76.7%) were of moderate quality. There were no randomized control trials and only one study included a well-being intervention designed specifically for MHCW. Most (67.4%) were quantitative-descriptive studies that compared well-being measures between minority and non-minority identifying healthcare workers. Common themes identified were burnout, job retention, job satisfaction, discrimination, and diversity. There were conflicting results regarding burnout rates in MHCW vs non-minority workers with some studies citing protective resilience and lower burnout while others reported greater burnout due to compounding systemic factors.

**Discussion:**

Our findings illuminate a lack of MHCW-specific well-being programs. The conflicting findings of MHCW well-being do not eliminate the need for supports among this population. Given the distinct experiences of MHCW, the development of policies surrounding diversity and inclusion, mental health services, and cultural competency should be considered. Understanding the barriers faced by MHCW can improve both well-being among the healthcare workforce and patient care.

## Introduction

Healthcare worker (HCW) well-being is of great public health importance as high rates of burnout are present throughout the medical field and are linked to poor patient care outcomes (1). Burnout can be defined as unsuccessfully managed chronic workplace stress that results in emotional exhaustion, job dissatisfaction, and reduced professional efficacy (2). Systemic factors such as workload, organizational structure, and access to support systems contribute to stress and burnout rates. Since the COVID-19 pandemic, well-being has taken center stage due to concerning levels of psychosocial strain throughout the healthcare workforce. A study by the United States Centers for Disease Control and Prevention (CDC) reported significantly higher burnout rates among health workers in 2022 (46%) compared to 2018 (32%) (3). In the global context, a report by the Qatar Foundation, World Innovation Summit for Health (WISH), in collaboration with the World Health Organization (WHO) found similar findings of healthcare worker burnout ranging from 41 to 52% (4). Other measures such as the number of poor mental health days, intent to change jobs, and harassment showed similar trends before and after the pandemic (3).

The negative impact on quality and costs of care associated with poor HCW well-being is also of significant concern (1). HCWs suffering from burnout may struggle to concentrate and be less detail-oriented, and more likely to make mistakes that can affect patient care and increase medical expenditures (5). Multiple studies have demonstrated a relationship between the onset of physician burnout and declining patient safety (6, 7). A systematic review by Hall et al. found that “poor [well-being] and moderate to high levels of burnout [were] associated, in the majority of studies reviewed, with poor patient safety outcomes.” (6) Increases in the frequency of hospital-acquired infections (8), mortality risk, and length of hospital stay have all been found to be associated with nurse burnout (9). A cost–benefit analysis of an institution-wide support program for nursing staff projected an estimated $1.81 million in annual hospital cost savings after implementation of the well-being intervention (10). Given these concerning findings, it is imperative to address well-being among HCWs.

Minority healthcare workers (MHCW), such as those self-identifying with racial/ethnic, sexual and gender, or migrant minority groups, face unique challenges that may affect their workplace associated well-being. In addition to traditional workplace pressures, MHCW may need to navigate systemic barriers such as structural racism, discrimination, and stereotyping. Further, they are more likely to work in underserved communities with limited resources (11). These circumstances may compound one another and contribute to differences in well-being compared to their non-minority counterparts. Although it is crucial to acknowledge the unique circumstances of this population, there is inconclusive evidence that MHCW experience worse well-being. Previous studies present conflicting evidence on burnout rates between minority and non-minority HCWs with some even suggesting that a minority background can be a protective factor (12).

There is limited data on interventions targeting the well-being of MHCW. Most well-being interventions are designed as one-size-fits-all solutions intended to apply to all workers. It may be important to implement targeted interventions that address the distinct needs of MHCW. By doing so, all HCWs, regardless of their sociodemographic backgrounds or identities, could feel supported and continue to provide high-quality care for their patients. The aim of this study was to identify and examine interventions specifically designed to support the well-being of MHCW. Additionally, the study analyzed existing literature on well-being outcomes and experiences comparing minority and non-minority HCWs.

## Methods

This systematic review was conducted between November 2023 and April 2024 in accordance with the Preferred Reporting Items for Systematic Reviews and Meta-Analyses (PRISMA, 2021) (13) guidelines. The study protocol was registered in the International Prospective Register of Systematic Reviews (PROSPERO, ID# CRD42023478339) prior to commencement of data collection.

### Search strategy

A comprehensive literature search was performed using the following databases: PubMed, Medline, Scopus, Embase, PsycINFO, Web of Science, CINAHL, ProQuest Dissertations and Theses Global, American Doctoral Dissertations, and Open Access Theses and Dissertations. Additional sources such as databases of gray literature, volumes of journals, reference lists of books, book chapters, systematic reviews were also searched. No time or study design restrictions were applied. The search was limited to availability in English. Multiple key terms and Boolean operators such as minority, underrepresented, healthcare worker, well-being, mental health, intervention, and program were used to target relevant papers. Results were limited to publications that included the search terms within their title or abstract text. The reference lists of eligible articles were also hand-searched to identify any additional publications. The detailed search strategy by database is included in Supplementary 1.

### Eligibility criteria

Published papers reporting original or secondary results of quantitative, qualitative, or mixed-methods research on the well-being of MHCW and targeted program interventions for MHCW were included. All study designs were considered inclusive of other systematic or scoping reviews in an effort to synthesize high-level evidence and reduce duplication of effort. We defined HCWs to be inclusive of physicians, pharmacists, physician assistants, nurses, hospital faculty, and their corresponding students or trainees. We chose to include early-career groups such as students as literature suggests early onset of burnout (14). Similarly, a broad characterization of minority was utilized to include racial/ethnic, gender, sexual, and migrant minority groups. These were defined within the geographic and cultural contexts in which the studies were conducted. The wide-ranging definitions were used capture more relevant data since published literature on this topic are relatively scarce. For inclusion in this review, articles must have included MHCW well-being outcomes or an intervention targeting the well-being of MHCW. Table 1 provides the detailed inclusion and exclusion criteria utilized to determine study eligibility.

**Table 1:** Inclusion and exclusion criteria.

Inclusion criteria	Exclusion criteria
Study population includes MHCW Reports on MHCW well-being outcomes or targeted, supportive interventions, directly or indirectly Full-text available, if applicable English language	Does not stratify findings by minority status Does not include outcomes related to well-being Interventions not aimed at well-being (e.g., educational) No abstract or full-text available for review Articles that are not evidence-based (e.g., opinion pieces, editorials, or commentaries) Duplicate paper included in a systematic or scoping review

### Study selection and data extraction

An online systematic review management system, Covidence (Covidence systematic review software, Veritas Health Innovation, Melbourne, Australia),^1^ was utilized for literature screening and data extraction. There were four reviewers, MM, TB, MC, and WH. Two independent reviewers conducted each title/abstract screening and full-text review. In cases of disagreement, consensus was acquired through discussion. At least one reviewer completed data extraction for selected articles. Extracted data included study title, author(s), date of publication, country, study design, type of HCW, type of MHCW, number of participants, attrition and response rate, well-being measures or interventions, well-being related primary and secondary outcomes (if applicable), and lessons learned.

### Data synthesis

Extracted information was exported from Covidence to Microsoft Excel (version 16.81). A spreadsheet was used for organization of the extracted data with a focus on relevant variables. Meta-analysis was not possible due to heterogeneity in the methodological features of the studies. Therefore, descriptive analysis of the included papers was conducted.

### Quality assessment

Included studies were categorized as quantitative (randomized, non-randomized, or descriptive), qualitative, mixed-methods, or systematic/scoping review. Due to the variety of study designs included in this review, two comprehensive critical appraisal tools, the Mixed Methods Appraisal Tool (MMAT, version 2018) (15, 16) and the Joanna Briggs Institute (JBI) Critical Appraisal Checklist for Systematic Reviews and Research Syntheses (2015) (17), were utilized. Two reviewers independently assessed all studies and disagreements were addressed through discussion to achieve consensus.

The MMAT is designed for the assessment of five study types: qualitative, quantitative randomized control, quantitative non-randomized, quantitative descriptive, and mixed-methods studies. There are two screening questions: (1) “Are there clear research questions?” and (2) “Do the collected data allow to address the research questions?” (15). The screening questions are followed by 25 appraisal items addressing quality criteria split into five sections corresponding to the specific study design, with each section having five questions. A total of five appraisal items are answered for all qualitative and quantitative study designs. A total of 15 questions are assigned for mixed-method studies as the specific two study designs included plus the mixed-methods-specific questions must be answered; however, the lowest score of the three categories is considered the overall quality. Response options to the series of questions include ‘yes’, ‘no’, or ‘cannot tell’. The full set of assessment questions can be found elsewhere (15). Quality was categorized as low (MMAT score, 0–2), moderate (MMAT score, 3–4), or high (MMAT score, 5).

The JBI tool was utilized for the quality appraisal of systematic reviews and scoping reviews. This tool has a total of 11 items with response options of ‘yes’, ‘no’, ‘unclear’, or ‘not applicable’. Overall quality was reported based on percentage of criteria as low (<40%), moderate (40–80%), or high (>80%).

## Results

### Study selection

**Figure 1:**
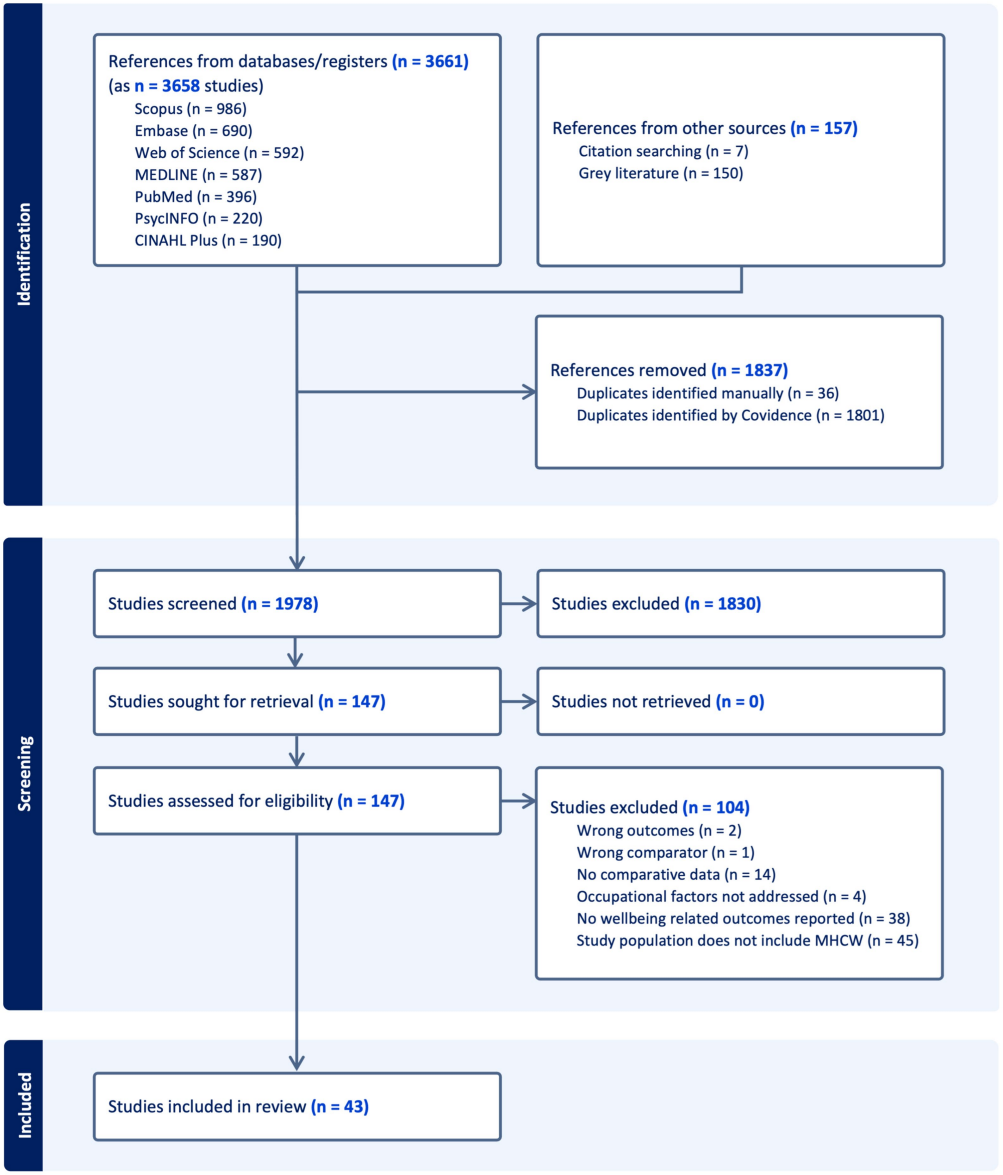
PRISMA diagram illustrating the search for relevant studies at different stages including identification, selection, and inclusion of the studies based on predefined criteria.

The initial search produced 3,815 records, of which 1,837 duplicates were removed (Figure 1). After screening of the available abstracts and titles by two independent reviewers, 147 studies were eligible for full-text review. Of these, 104 were excluded for various reasons (detailed in Figure 1) resulting in 43 included studies.

### Study characteristics

All 43 included studies were written in English and published between 2006 and 2023. Most of the studies (67.4%) utilized quantitative descriptive methods, 7 (16.3%) qualitative, 3 (7.0%) systematic or scoping reviews, 2 (4.7%) quantitative non-randomized, and 2 (4.7%) were mixed-methods studies. No studies utilized a randomized design. Geographic location was mostly the United States (81.4%) followed by Canada (7.0%) and the United Kingdom (4.7%). Other countries included Germany, Israel, and Malaysia. There was wide variability in the types of healthcare professionals included: physicians, medical trainees (fellows, residents, and students), nurses, health department employees, physician scientists, and other clinical-based students. A majority of studies defined minority status by race/ethnicity. Table 2 summarizes the key characteristics of the included studies.

**Table 2:** Characteristics of included studies.

Characteristic	N (Total *n* = 43)	%
Study design		
Quantitative descriptive	29	67.4
Qualitative	7	16.3
Quantitative non-randomized	2	4.7
Mixed-methods	2	4.7
Systematic/scoping review	3	7.0
Country		
United States	35	81.4
Canada	3	7.0
United Kingdom	2	4.7
Germany	1	2.3
Israel	1	2.3
Malaysia	1	2.3
Type of Healthcare Worker		
Physicians	13	30.2
Medical trainee (fellow/resident/student)	11	25.6
Nurses	5	11.6
Doctoral students	1	2.3
Health department employees	1	2.3
PhD/physician scientists	1	2.3
Physician associate students	1	2.3
Residency program directors	1	2.3
Student registered nurse anesthetists	1	2.3
Multiple	7	16.3
Multiple (students only)	1	2.3

Studies measured several well-being domains such as burnout, emotional exhaustion, depersonalization, job satisfaction, intent to leave, depression, anxiety, and discrimination. Many authors created their own questionnaires or adapted already existing ones. Burnout was the most commonly assessed well-being outcome. Multiple validated and non-validated tools were utilized in assessing burnout; 10 studies utilized the Maslach Burnout Inventory (MBI) (18) and 5 used the Copenhagen Burnout Inventory (CBI) (19). All authors used paper-and-pencil or web-based/electronic self-report questionnaires and nine studies used semi-structured interviews to identify qualitative themes. A majority of studies used convenience sampling to identify participants, however we included data from 4 large national systematic surveys that compare well-being measures for racial/ethnic minority and non-minority HCWs.

### Study quality

The MMAT was used to evaluate 40 studies (Table 3). All of these had a response of ‘yes’ to the two screening questions, which were not included in Table 3. MMAT questions 2.1 to 2.5 were also not included as no studies met the design criteria. All primary studies met more than half of the quality criteria of MMAT and used an appropriate sample frame to address the target population. However, the adequacy of the sample size and the use of valid methods was unclear for several studies. Two systematic reviews and 1 scoping review used the JBI appraisal tool. Table 4 shows appraisal results of JBI-evaluated studies. Overall, 76.7% of the included studies were of moderate methodological quality and 23.3% were of high quality. No studies were rated as low quality.

**Table 3:** Appraisal of quantitative, qualitative, and mixed-method studies using MMAT.

Study design; Author (year)	Assessment criteria					
1. Qualitative	1.1	1.2	1.3	1.4	1.5	Quality
Boateng et al. (2019) (26)	Y	Y	Y	Y	Y	High
Cedeño et al. (2023) (20)	Y	Y	Y	N	Y	Moderate
Chilakala et al. (2022) (21)	Y	Y	Y	Y	Y	High
Keshet and Popper-Giveon (2016) (22)	Y	Y	0	Y	Y	Moderate
Klingler and Marckmann (2016) (23)	Y	Y	0	Y	Y	Moderate
Nfonoyim et al. (2021) (24)	Y	Y	Y	Y	Y	High
Rivera (2018) (25)	N	Y	Y	Y	Y	Moderate
3. Quantitative non-randomized	3.1	3.2	3.3	3.4	3.5	Quality
Daley et al. (2006) (56)	Y	Y	N	N	Y	Moderate
Jaishankar et al. (2021) (27)	N	Y	Y	Y	Y	Moderate
4. Quantitative descriptive	4.1	4.2	4.3	4.4	4.5	Quality
Armstrong and Reynolds (2020) (28)	N	Y	Y	N	Y	Moderate
Bazargan-Hejazi et al. (2023) (65)	Y	Y	Y	N	Y	Moderate
Burns et al. (2021) (29)	Y	Y	Y	0	Y	Moderate
Carthon et al. (2021) (30)	Y	Y	Y	0	Y	Moderate
Chin et al. (2016) (50)	Y	N	Y	Y	Y	Moderate
Doede (2017) (31)	Y	Y	Y	0	Y	Moderate
Douglas et al. (2021) (51)	Y	Y	Y	N	Y	Moderate
Dyrbye et al. (2007) (52)	Y	Y	Y	Y	Y	High
Dyrbye et al. (2006) (32)	Y	N	Y	Y	Y	Moderate
Evans et al. (2021) (33)	Y	Y	Y	N	Y	Moderate
Ey et al. (2013) (57)	Y	Y	Y	N	Y	Moderate
Frias and Yuen (2021) (34)	Y	Y	Y	N	Y	Moderate
Garcia et al. (2020) (12)	Y	Y	Y	Y	Y	High
Glymour et al. (2004) (35)	Y	Y	Y	Y	N	Moderate
Graham-Brown et al. (2021) (36)	Y	Y	Y	Y	Y	High
Greenberg et al. (2022) (37)	Y	Y	Y	N	Y	Moderate
Khan et al. (2021) (38)	Y	Y	Y	Y	Y	High
Mitchell et al. (2022) (39)	Y	Y	Y	N	N	Moderate
Nunez-Smith et al. (2009) (66)	Y	N	Y	N	Y	Moderate
Obichi et al. (2023) (67)	Y	Y	Y	N	Y	Moderate
Odei and Chino (2021) (40)	Y	Y	Y	N	N	Moderate
Padela et al. (2016) (41)	Y	Y	Y	N	Y	Moderate
Perina et al. (2018) (68)	Y	Y	Y	N	Y	Moderate
Pillado et al. (2023) (42)	Y	Y	Y	Y	Y	High
Primack et al. (2010) (43)	Y	N	Y	N	Y	Moderate
Psenka et al. (2020) (44)	Y	Y	Y	N	Y	Moderate
Rhead et al. (2020) (45)	Y	Y	N	Y	N	Moderate
Serrano et al. (2023) (46)	Y	Y	Y	N	Y	Moderate
Yoon et al. (2010) (47)	Y	Y	Y	Y	N	Moderate
5. Mixed-methods	5.1	5.2	5.3	5.4	5.5	Quality
Doyle et al. (2021) (48)	Y	0	0	Y	Y	Moderate
Eliason et al. (2018) (49)	Y	Y	Y	N	N	Moderate

MMAT scores of 0–2 represent low quality, 3–4 moderate quality, and 5 is high quality. 0 = cannot tell; N = no; Y = yes.

**Table 4:** Appraisal of systematic and scoping reviews using JBI critical appraisal checklist for systematic reviews and research syntheses.

Assessment criteria	Study author (year)		
	Abrahim and Holman (2023) (53)	Alvandi and Davis (2023) (55)	Lawrence et al. (2022) (54)
1. Is the review question clearly and explicitly stated?	Y	Y	Y
2. Were the inclusion criteria appropriate for the review question?	Y	Y	Y
3. Was the search strategy appropriate?	Y	Y	Y
4. Were the sources and resources used to search for studies adequate?	Y	Y	Y
5. Were the criteria for appraising studies appropriate?	N	Y	Y
6. Was critical appraisal conducted by two or more reviewers independently?	N	Y	Y
7. Were there methods to minimize errors in data extraction?	N	Y	Y
8. Were the methods used to combine studies appropriate?	Y	Y	Y
9. Was the likelihood of publication bias assessed?	N	Y	Y
10. Were recommendations for policy and/or practice supported by the reported data?	Y	Y	Y
11. Were the specific directives for new research appropriate?	Y	Y	Y
Overall Appraisal	Include	Include	Include
Quality	Moderate	High	High

N = no; Y = yes.

### Study outcomes

#### Well-being in minority versus non-minority HCWs

Most qualitative studies (85.7%) did not have non-minority comparison groups, however all identified negative experiences for MHCW (20–25). The qualitative themes identified included exposure to microaggressions, institutional ostracizing, tense working environment, racial isolation, lack of culturally diverse mentors, stereotypical or offensive attitude from patients, unprofessional encounters from peers, pressure to prove themselves as a result of negative experiences, and fear of being othered. Experiences of microaggression and discrimination were reported by both racial/ethnic and gender/sexual MHCW.

A majority of studies (*n* = 29, 67.4%) compared the well-being of minority and non-minority HCWs either qualitatively or quantitively. Of these, the vast majority (82.8%) noted some worse outcomes in the MHCW population (26–49). One study found no significant overall difference in burnout by gender or ethnicity (50). Three studies reported better well-being among MHCW in comparison to their non-minority counterparts (12, 51, 52). Garcia et al. (12) reported lower adjusted odds of burnout among minority racial/ethnic groups in comparison to non-Hispanic white participants (Hispanic/Latinx physicians, odds ratio [OR] = 0.63, 95% confidence interval [CI] [0.47, 0.86]; non-Hispanic Black physicians, OR = 0.49, 95% CI [0.30, 0.79]). This study was a secondary analysis of survey data from 4,424 physicians, using MBI to assess burnout. Authors noted several limitations including a much lower response rate for minority physicians and the utilization of the American Medical Association’s Physician Masterfile dataset to identify minority physicians, which lacked comprehensive racial/ethnic information. Additionally, Abrahim and Holman (53) conducted a scoping review of literature on the well-being of racial and ethnic minority nurses during the COVID-19 pandemic. Two studies in their review documented greater anxiety among white nurses, but contained relatively small, predominantly white, unrepresentative samples. The authors concluded that “findings for the nurses of color may not be reliable because the samples included few racial and ethnic minority nurses.” (53).

In regards to overall burnout scores, two studies found no significant difference between minority and non-minority medical students (28, 32). However, one of these studies (28) (*n* = 162) showed significantly higher rates of personal burnout among racial/ethnic minority medical students (*p* = 0.001). The second study (32) surveyed medical students (*n* = 545) and although there were similarly no overall differences in burnout, emotional exhaustion, or depersonalization, minority medical students had a significantly lower sense of personal accomplishment (42% vs. 28%; *p* = 0.02). Also of note, minority students were less likely to respond to the survey (37% vs. 50%; *p* < 0.001).

A systematic review by Lawrence et al. (54) that focused on the racial/ethnic differences in burnout rates had inconclusive findings, and recommended increased evaluation and focus on systemic factors that may be at play. Three of the 16 studies in this review did not include HCWs. Additionally, Lawrence et al. noted that their findings were nuanced and several of the included studies had methodological issues.

The majority of studies used convenience sampling to identify participants, but our sample also included data from four large national systematic surveys that compared well-being measures for racial minority and non-minority HCWs. The largest of these (31) (*n* = 27,953) was from the National Sample Survey of Registered Nurses. It found that Asians had lower odds (*p* < 0.001) of job dissatisfaction and having changed jobs (*p* < 0.001) compared to white counterparts, while Black and Hispanic participants showed no significant association. The authors concluded that race/ethnicity was a predictor of job satisfaction and turnover and Asian nurses showed more positive outcomes than white nurses, while Black and Hispanic individuals showed significantly worse outcomes. Another large national survey (30) (*n* = 14,778) reporting on the data from RN4CAST-U.S found that Black nurses reported greater job dissatisfaction (*p* < 0.001) and intent to leave within a year (*p* < 0.001) in comparison to white nurses. A national training survey from the UK General Medical Council of 627 renal medicine physicians similarly suggested that racial/ethnic minority medical trainees reported higher burnout rates than white trainees (36).

However, data from a national physician survey (*n* = 3,096) from the American Board of Family Medicine (ABFM) Family Medicine Continuing Certification Examination Registration questionnaire showed that minority physicians were significantly less likely to report depersonalization, both as a binary variable (*p* = 0.03) and continuous variable (*p* < 0.001), less likely to report emotional exhaustion as a continuous variable (*p* = 0.04), but equally likely to report emotional exhaustion (*p* = 0.09) (51). Minority physicians were more likely to work in counties with higher diversity index and authors concluded that working in racially and ethnically diverse environments could be a mediating factor resulting in a lower frequency of emotional exhaustion and feelings of depersonalization (51).

Among studies that compared outcomes between men and women (*n* = 16), 68.8% found worse well-being among women HCWs. Yoon et al. (47) explored conflict as a correlate of burnout among HCWs. No association was found between conflict over treatment decisions and race/ethnicity, but there was a significant positive association among women physicians. Reasons for this association are not well understood, but Yoon et al. (47) suggest that female patients are more likely to choose female physicians and more willing to voice disagreements with physicians of the same gender. Five studies specifically investigated gender differences in burnout. Two of these studies did not find any difference in burnout among male and female HCWs (38, 50). One study found that male renal trainees reported higher burnout rates than women colleagues (36). However, a systematic review of 11 studies by Alvandi and Davis (55) showed that female HCWs reported greater burnout.

Sexual minority HCWs were examined in four studies. Key findings include discomfort in ‘coming-out’ in the workplace (49), being socially excluded (25), and greater odds of depressive or anxiety symptoms than heterosexual counterparts (33).

#### Well-being interventions

Only one study, by Daley et al. (56) included a specific MHCW-focused well-being intervention. They examined the change in retention rate among health center faculty after the implementation of the Junior Faculty Development Program for minority-identifying faculty which provided development workshops, counseling, and mentoring. There was a non-significant increase in retention rate of 15% among minority-identifying faculty in academic medicine. One other study by Ey et al. (57) included an intervention that was not tailored to the MHCW population. They evaluated a Resident Wellness Program that provided free, on-site counseling for all medical trainees regardless of minority status. Findings indicated that MHCW were significantly less likely to utilize the program.

Details of study population, measures, outcomes, and lessons learned of all included studies are summarized in Supplementary 2.

## Discussion

Contrary to our expectations, there were few publications on supportive interventions that specifically target MHCW. We found only one published MHCW-specific intervention, a development and mentorship program which showed no significant association with retention rate (56). Instead, findings from this review suggest that creating a safe work environment and empowering MHCW to participate in well-being interventions may be important. One study (57) showed that MHCW were less likely that their non-minority counterparts to utilize their well-being program. Several studies found discomfort among HCWs in receiving support, and that time away from work may be a potential barrier to utilizing well-being programs (57, 58). These barriers may be greater for MHCW experiencing discrimination and other systemic challenges, who may want to avoid any additional discomfort in the workplace.

To our knowledge, this is the first systematic review to comprehensively explore the well-being of MHCW. We aimed to cover a broad topic area as we felt that any narrowing of the well-being definition may result in selection bias. The search included a broad range of search terms with minimal limitations. All study designs were included and no studies were excluded based on quality. Uniformity in the critical appraisal process was prioritized by usage of the MMAT which encompassed most study designs. This important review provided context and awareness to how intersectional factors (e.g., race, gender, sexual orientation) affects the well-being of the minority-identifying healthcare workforce, highlighting the importance of inclusivity and equity in the workplace and providing evidence-based syntheses for policymakers to improve the well-being of MHCW.

Consistent with previous literature, we found inconsistencies in well-being outcomes among MHCW. There are potential reasons for these discrepant findings. It is important to consider that minority populations may express stress differently which could affect scores on well-being measures. The validity of previously established stress models has been questioned for minority populations. Ivey and Gauch (59) demonstrated that “minority communities have different distributions of emotions than the general population” and that existing models may not be representative as they are not trained with minority-specific data. Singh et al. (60) found that chronic stress among individuals who experience continuous discrimination leads to emotional dysregulation and emotional suppression. They argued that the “impact of any instance of social isolation, discrimination, and bias is directly responsible for suppression of emotional expression” (60) whether that be negative or positive responses. This potential reporting bias should be considered in interpreting our findings.

Notably, larger, well-represented national surveys tended to find higher rates of burnout among MHCW. It also appeared that burnout among MHCW can be modulated by a variety of professional and environmental factors. For example, working in racially and ethnically diverse environments was found to be a mediating factor reducing burnout among minority family physicians (51). MHCW may feel less minoritized in settings that promote diversity. Similarly, in a survey of 519 oncologists, 48 minority radiation oncologists reported greater burnout rates than non-minority, but minority medical oncologists reported lower burnout rates than non-minority respondents. This suggests a potential influence of differences in work environment on burnout (40).

Psychological distress can be cumulative over the life course and can also be compounded by the presence of multiple stressors. For example, identifying with more than one type of minority or identifying with an ‘invisible’ minority group may be associated with worse outcomes. Those identifying as sexual minorities, in particular, are sometimes able to make a ‘choice’ about coming-out to colleagues and patients, as opposed to those with racial/ethnic minority status which may be more visibly apparent. This could worsen well-being among the LGBTQ+ community as they internalize negative feelings. Future studies should control for or evaluate the differences associated with a particular minority group, however this is understandably challenging given the concept of intersectionality in identity. There is a complex interplay of various facets of identity, such as ethnicity, gender, sexuality, professional seniority, etc., that do not exist in isolation but rather intersect in various ways to shape an individual’s context (61).

There are multiple benefits to an inclusive and supportive work environment in promoting well-being among MHCW. Wolfe (62) theorizes that LGBTQ+ healthcare professionals, similar to other minority-identifying populations, encounter incongruence between their personal and professional identities, and thus have differential experiences with mental distress and burnout. They also call for “intersectional actions that recognize and mitigate spaces of inequality that constrain the benefit marginalized professionals receive from improvement efforts” (62) such as interventions tailored to specific minority group needs. Brown et al. (63) emphasize that medical education diversity goals are only attainable “when inclusion and equity are on the table as well” since a supportive work environment must address systemic inequalities in order to promote well-being among the workforce. Prioritizing diversity and inclusion policies requires a systemic approach of stakeholder collaboration, strategy evaluation, and community engagement (64). This will also have a direct impact on the retention of MHCW. Additionally, a healthcare workplace that closely represents the community it serves will improve the quality of care (64).

## Limitations

This study has some limitations. There was methodological heterogeneity in the types of well-being outcomes and the measurement tools included in studies. However, there was less heterogeneity in the study populations. Studies also utilized different definitions of “minority” with most focusing on racial/ethnic minorities, while others included immigration status and religious affiliation. This prevented aggregation or quantitative comparisons of results. Furthermore, findings from different countries were included in this review without consideration of the cultural, political, and economic contexts surrounding healthcare. Therefore, the results should be interpreted in context.

## Conclusion

There is paucity of published evidence on supportive interventions to address MHCW well-being. The results of our review do not fully support the need for well-being programs tailored solely to MHCW. However, we could only find one study specifically supporting MHCW’s. It is possible that systematic barriers such as discrimination are preventing MHCW’s participation in support programs. Given the complex and intersectional nature of identity, it is understandable that there is no one “size” approach even among a particular population of MHCW. Rather, a broad public health approach should be considered to mitigate the negative health outcomes and improve utilization of support programs, including the development and implementation of policies surrounding diversity and inclusion, mental health services, and cultural competency. By increasing focus on the barriers to well-being faced by MHCW, the well-being of the entire healthcare workforce could be improved and subsequently translate into better patient care. We recommend future research on MHCW utilizing validated well-being measures and incorporating a wider geographical variation beyond North America and Europe, especially from underrepresented countries.

## Data Availability

The raw data supporting the conclusions of this article will be made available by the authors, without undue reservation.

## Footnotes

^1^ www.covidence.org
