# Supplementary material for: Systematic review of well-being interventions for minority healthcare workers
Source: Front Med (Lausanne). 2025 Feb 21;12:1531090. doi: 10.3389/fmed.2025.1531090 (PMC11885290; doi:10.3389/fmed.2025.1531090)
Supplement: Supplementary file 1 [file Data_Sheet_1.docx]

**Supplement 1:** Detailed Search Strategy

PubMed

- Date of search: November 08, 2023
- Search Strategy: ("minority"[Title/Abstract] OR "underrepresented"[Title/Abstract] OR "marginalized"[Title/Abstract]) AND ("health personnel"[Title/Abstract] OR "healthcare personnel"[Title/Abstract] OR "health professional"[Title/Abstract] OR "healthcare professional"[Title/Abstract] OR "health worker"[Title/Abstract] OR "healthcare worker"[Title/Abstract] OR "physician"[Title/Abstract] OR "doctor"[Title/Abstract] OR "clinician"[Title/Abstract] OR "nurse"[Title/Abstract] OR "dentist"[Title/Abstract] OR "healthcare provider"[Title/Abstract]) AND ("well-being"[Title/Abstract] OR "wellbeing"[Title/Abstract] OR "mental health"[Title/Abstract] OR "stress"[Title/Abstract] OR "anxiety"[Title/Abstract] OR "depression"[Title/Abstract] OR "burnout"[Title/Abstract] OR "turnover"[Title/Abstract]) AND ("intervention"[Title/Abstract] OR "training"[Title/Abstract] OR "education"[Title/Abstract] OR "program"[Title/Abstract] OR "mental health support"[Title/Abstract] OR "resilience training"[Title/Abstract] OR "psychological support"[Title/Abstract] OR "stress management"[Title/Abstract] OR "burnout prevention"[Title/Abstract] OR "second victim program"[Title/Abstract] OR "psychological first aid"[Title/Abstract] OR "debriefing"[Title/Abstract] OR "suicide prevention"[Title/Abstract] OR "resilience"[Title/Abstract] OR "satisfaction"[Title/Abstract])
- Field: Title/abstract
- Filters: English language
- n findings: 396

MEDLINE-Ovid

- Date of search: November 09, 2023
- (("minority" or "underrepresented" or "marginalized") and ("health personnel" or "healthcare personnel" or "health professional" or "healthcare professional" or "health worker" or "healthcare worker" or "physician" or "doctor" or "clinician" or "nurse" or "dentist" or "healthcare provider") and ("well-being" or "wellbeing" or "mental health" or "stress" or "anxiety" or "depression" or "burnout" or "turnover")).mp. and ("intervention" or "training" or "education" or "program" or "mental health support" or "resilience training" or "psychological support" or "stress management" or "burnout prevention" or "second victim program" or "psychological first aid" or "debriefing" or "suicide prevention" or "resilience" or "satisfaction").ab,kw,ti.
- Field: Title/Abstract/Keyword
- Limiters: English available
- n findings: 587

Scopus

- Date of search: November 07, 2023
- TITLE-ABS ( ( minority OR underrepresented OR marginalized ) AND ( "health personnel" OR "healthcare personnel" OR "health professional" OR "healthcare professional" OR "health worker" OR "healthcare worker" OR "physician" OR "clinician" OR "nurse" OR "healthcare provider" ) AND ( "well-being" OR "wellbeing" OR "mental health" OR "stress" OR "anxiety" OR "depression" OR "burnout" OR "turnover" ) AND ( "intervention" OR "training" OR "education" OR "program" OR "mental health support" OR "resilience training" OR "psychological support" OR "stress management" OR "burnout prevention" OR "second victim program" OR "psychological first aid" OR "debriefing" OR "suicide prevention" ) ) AND ( LIMIT-TO ( DOCTYPE , "ar" ) ) AND ( LIMIT-TO ( LANGUAGE , "English" ) )
- Field: Title/Abstract
- Limiters: English language, Publication type article
- n findings: 986

APA PsycINFO

- Date of search: November 08, 2023
- Search Strategy: AB ("minority" OR "underrepresented" OR "marginalized" OR "migrant" OR "foreign-born" OR “foreign medical graduate”) AND ("health personnel" OR "healthcare personnel" OR "health professional" OR "healthcare professional" OR "health worker" OR "healthcare worker" OR "physician" OR "doctor" OR "clinician" OR "nurse" OR "dentist" OR "healthcare provider") AND ("well-being" OR "wellbeing" OR "mental health" OR "stress" OR "anxiety" OR "depression" OR "burnout" OR "turnover") AND ("intervention" OR "training" OR "education" OR "program" OR "mental health support" OR "resilience training" OR "psychological support" OR "stress management" OR "burnout prevention" OR "second victim program" OR "psychological first aid" OR "debriefing" OR "suicide prevention" OR “resilience” OR "satisfaction”)
- Field: Abstract
- Limiters: English language
- n findings: 220

CINAHL Plus

- Date of search: November 08, 2023
- Search Strategy: AB ("minority" OR "underrepresented" OR "marginalized" OR "migrant" OR "foreign-born" OR “foreign medical graduate”) AND ("health personnel" OR "healthcare personnel" OR "health professional" OR "healthcare professional" OR "health worker" OR "healthcare worker" OR "physician" OR "doctor" OR "clinician" OR "nurse" OR "dentist" OR "healthcare provider") AND ("well-being" OR "wellbeing" OR "mental health" OR "stress" OR "anxiety" OR "depression" OR "burnout" OR "turnover") AND ("intervention" OR "training" OR "education" OR "program" OR "mental health support" OR "resilience training" OR "psychological support" OR "stress management" OR "burnout prevention" OR "second victim program" OR "psychological first aid" OR "debriefing" OR "suicide prevention" OR “resilience” OR "satisfaction”)
- Field: Abstract
- Limiters: English language
- n findings: 190

EMBASE

- Date of search: November 08, 2023
- Search Strategy: ('minority':ti,ab,kw OR 'underrepresented':ti,ab,kw OR 'marginalized':ti,ab,kw OR 'migrant':ti,ab,kw OR 'foreign-born':ti,ab,kw OR 'foreign medical graduate':ti,ab,kw) AND ('health personnel':ti,ab,kw OR 'healthcare personnel':ti,ab,kw OR 'health professional':ti,ab,kw OR 'healthcare professional':ti,ab,kw OR 'health worker':ti,ab,kw OR 'healthcare worker':ti,ab,kw OR 'physician':ti,ab,kw OR 'doctor':ti,ab,kw OR 'clinician':ti,ab,kw OR 'nurse':ti,ab,kw OR 'dentist':ti,ab,kw OR 'healthcare provider':ti,ab,kw) AND ('well-being':ti,ab,kw OR 'wellbeing':ti,ab,kw OR 'mental health':ti,ab,kw OR 'stress':ti,ab,kw OR 'anxiety':ti,ab,kw OR 'depression':ti,ab,kw OR 'burnout':ti,ab,kw OR 'turnover':ti,ab,kw) AND ('intervention':ti,ab,kw OR 'training':ti,ab,kw OR 'education':ti,ab,kw OR 'program':ti,ab,kw OR 'mental health support':ti,ab,kw OR 'resilience training':ti,ab,kw OR 'psychological support':ti,ab,kw OR 'stress management':ti,ab,kw OR 'burnout prevention':ti,ab,kw OR 'second victim program':ti,ab,kw OR 'psychological first aid':ti,ab,kw OR 'debriefing':ti,ab,kw OR 'suicide prevention':ti,ab,kw OR 'resilience':ti,ab,kw OR 'satisfaction':ti,ab,kw) AND [english]/lim
- Field: Abstract, Title, Keywords
- Limiters: English language
- n findings: 690

Web of Science

- Date of search: November 08, 2023
- TS=( (("minority" OR "underrepresented" OR "marginalized" OR "migrant" OR "foreign-born" OR “foreign medical graduate”) AND ("health personnel" OR "healthcare personnel" OR "health professional" OR "healthcare professional" OR "health worker" OR "healthcare worker" OR "physician" OR "doctor" OR "clinician" OR "nurse" OR "dentist" OR "healthcare provider") AND ("well-being" OR "wellbeing" OR "mental health" OR "stress" OR "anxiety" OR "depression" OR "burnout" OR "turnover") AND ("intervention" OR "training" OR "education" OR "program" OR "mental health support" OR "resilience training" OR "psychological support" OR "stress management" OR "burnout prevention" OR "second victim program" OR "psychological first aid" OR "debriefing" OR "suicide prevention" OR “resilience” OR "satisfaction”)))
- Field: Topic (Abstract, Title, Keywords)
- Limiters: English language
- n findings: 592

Grey Literature

- n findings: 150

Citation Searching

- n findings: 7
