# Supplementary material for: Systematic review of well-being interventions for minority healthcare workers
Source: Front Med (Lausanne). 2025 Feb 21;12:1531090. doi: 10.3389/fmed.2025.1531090 (PMC11885290; doi:10.3389/fmed.2025.1531090)
Supplement: Supplementary file 2 [file Supplementary_file_2.docx]

**Supplement 2:** Methodology and Findings of Included Studies

| Study Author (Year); Country | Study Type (n= Analytic Sample) | Type of HCW | Type of Minority | Measurement Tools or Intervention | Main Outcomes | Lessons Learned |
| --- | --- | --- | --- | --- | --- | --- |
| Abrahim and Holman (2023)^1^; US | Scoping Review | Registered nurses; 7 articles | Racial/ethnic | Various: Generalized Anxiety Disorder-7 (GAD-7),^2^ Patient Health Questionnaire-2 (PHQ-2),^3^ researcher-created questionnaires, semi-structured interviews | - Two studies noted white nurses to be more likely to report anxiety^4,5^ - Inconsistent findings related to depression with one reporting no difference^4^ and one reporting greater depression among White nurses^5^ - In other studies that included heterogenous participants, no difference in well-being was noted. | There were few significant differences by race/ethnicity. In studies that included only nurses, white nurses reported worse outcomes. |
| Alvandi and Davis (2023)^6^; US | Systematic Review | Medical faculty; 17 articles (n=7447) | Gender | Various: survey, focus groups, Maslach Burnout Inventory (MBI)^7^ | - One study found that women physicians reported a higher level of depersonalization and exhaustion.^8^ - Two studies noted increased burnout among females.^8,9^ | Burnout varied by gender. |
| Armstrong and Reynolds (2020)^10^; US | Quantitative descriptive | Medical students (n=162) | Non-white (43%), underrepresented minority (URM, 28.4%) and gender (women 64%) | Survey: Copenhagen Burnout Inventory (CBI)^11^ plus additional questions | - No statistically significant difference in overall burnout between minority and non-minority students. - Black students had significantly greater personal burnout scores than other races (p=0.01). - Women were more likely to experience work-related burnout (p=0.03). | Medical students, specifically Black students and women, may experience greater rates of burnout. |
| Bazargan-Hejazi et al. (2023)^12^; US | Quantitative descriptive | Students enrolled in nursing, medicine, or allied health programs (n=137) | Non-white (96.4% of which 25.0% Hispanic, 42.4% Black or African American, 22.0% Asian/Pacific Islander, 10.6% other non-white) and gender (women 70%) | Survey: 92-item anonymous questionnaire assessing positive emotion, engagement, relationships, meaning, and accomplishment (PERMA) | - Overall high levels of PERMA well-being scores among this diverse sample. - Women scored higher on positive emotion in comparison to men. | This study measured well-being among a majority racial and ethnic minority sample and found overall good well-being. There was no non-minority comparison group. |
| Boateng et al. (2019)^13^; Canada | Qualitative | Registered nurses and registered practical nurses (n=70) | Racial/ethnic (54.3%), gender (women 90%), and immigrant (60%) | Interview: One-on-one, semi-structured in-depth interviews | - Over half of ethnic minority nurses reported experiencing discrimination in the workplace and increase emotional exhaustion in comparison to their white counterparts. - White nurses reported greater job satisfaction. - Both minority and non-minority nurses equally reported organizational well-being supports. | Differences in well-being exist between minority and non-minority nurses, thus organizations should “embrace diversity in specific and meaningful ways that provide adequate support for [those] who are more vulnerable in multiple facets of wellness.”^13^ |
| Burns et al. (2021)^14^; Canada | Quantitative descriptive | Full-time clinical faculty members (n=419) | URM (29.8%) and disability (3.4%) | Survey: Stanford Professional Fulfillment Index (PFI)^15^ | - Unprofessionalism experiences, female gender, and disability were associated with lower PFI scores. | Minority faculty experienced lower professional fulfillment, and there is a need to implement strategies to address contributing factors. |
| Carthon et al. (2021)^16^; US | Quantitative descriptive | Nurses (n=14,778) | Racial/ethnic, specifically Black (5.8%) | Survey: RN4CAST-U.S. Study on job outcomes, intent to leave, and job satisfaction | - Black nurses were more likely to be employed in long term care. - Black nurses reported greater job dissatisfaction (p<0.001) and intent to leave within a year (p<0.001) in comparison to white nurses. | Black nurses reported greater job dissatisfaction and intent to leave in comparison to white nurses. |
| Cedeño et al. (2023)^17^; US | Qualitative | Medical students (n=12) | Race/ethnicity, specifically those self-identifying as Black, Indigenous, and People of Color (BIPOC, 100%), and gender (women 75%) | Interview: 4 focus group discussions and 1 individual interview | - Participants who rotated in rural, predominately white settings frequently reported feelings of racial isolation. - All participants reported experiencing at least 1 microaggression during clinical rotations, however had an overall positive learning experience. | Medical schools should acknowledge that BIPOC-identifying students may encounter discrimination and microaggressions during training at some sites, and perhaps should prioritize the inclusion of diverse sites. |
| Chilakala et al. (2022)^18^; US | Qualitative | Physicians (n=20) | Black (100%) and gender (women 100%) | Interview: Semi-structured, in-depth interviews | - Two themes identified through thematic analysis of interviews: (1) experiencing and managing micro/macroaggressions and biases in the workplace and (2) strategies to overcome experiences of intersectional discrimination. - Participants described numerous sources of discrimination, yet ineffective methods to mitigate its impact. | Black female physicians experienced poor well-being outcomes as a result of discrimination. There is a need to establish and evaluate interventions. |
| Chin et al. (2016)^19^; Malaysia | Quantitative descriptive | Medical students (n=452) | Ethnicity (Malay 43.1%, Chinese 38.5%, Indian 14.6%, others 3.8%) and gender (women 63.1%) | Survey: CBI^11^ | - Overall burnout prevalence was 67.9% with no significant difference by gender and ethnicity (p>0.05). | There was no significant association of burnout with gender and ethnicity. |
| Daley et al. (2006)^20^; US | Quantitative non-randomized | Junior faculty defined as full-time faculty at the assistant-professor level (n=112) | URM (13.4%) | Intervention: National Center for Leadership in Academic Medicine (NCLAM) program:   1. Twelve development workshops 2. Structured seven-month one-on-one mentorship program 3. Two-hour academic performance counseling session 4. Professional development project | - Program targeting minority faculty members, however anyone regardless of race/ethnicity was allowed to participate. - The retention rate of URM faculty at the School of Medicine increased from 58% to 80% after implementation of the program. Similarly, URM faculty in academic medicine saw a 15% increase in retention rates. However, neither of these changes in retention rate were statistically significant. | The implementation of a focused faculty development and mentorship program is associated with a nonsignificant increase in retention rate. |
| Doede (2017)^21^; US | Quantitative descriptive | Nurses (n=27,953) | Racial/ethnic (Black 5.7%, Hispanic 3.8%, Asian 6.0%) | Survey: 2008 National Sample Survey of Registered Nurses (NSSRN)^22^ | - Multivariable-adjusted regression models showed that Asians had lower odds (p<0.001) of job dissatisfaction and having changed jobs (p<0.001) when compared to white counterparts, while Black and Hispanic participants showed no significant association. - Black, Hispanic, and Asian nurses had significant differences when compared to white nurses in regards to intending to quit which Black and Hispanic nurses having higher odds and Asian nurses having lower odds. | Race/ethnicity is a predictor of job satisfaction and turnover. Asian nurses showed more favorable outcomes than white nurses, while Black and Hispanic showed worse outcomes. |
| Douglas et al. (2021)^23^; US | Quantitative descriptive | Physicians (family medicine, n=3,096) | Racial/ethnic groups underrepresented in medicine (URiM, 15%) | Survey: 2017 American Board of Family Medi- cine (ABFM) Family Medicine Continuing Certification Examination Registration questionnaire and 2017 National Graduate Survey (NGS) | - Physicians URiM were more likely to practice in counties with higher diversity indexes in comparison to non-minority physicians. - In comparison to their non-minority counterparts, physicians URiM were significantly less likely to report depersonalization as a binary (p=0.03) and continuous variable (p<0.001), less likely to report emotional exhaustion as a continuous variable (p=0.04), but similarly likely to report binary emotional exhaustion (p=0.09). - After inclusion of covariates and mediators such as age, sex, medical degree type, practicing obstetrics, providing pediatric care and adult hospital care, and county diversity index, URiM status remained a significant predictor only for depersonalization (p=0.002). | Working in racially and ethnically diverse environments was found to be a mediating factor resulting in a lower frequency of emotional exhaustion and feelings of depersonalization among family physicians URiM. There was overall lower burnout among minority physicians vs non-minority physicians. |
| Doyle et al. (2021)^24^; US | Mixed-methods | PhD researchers and physician-scientists (n=196) | Racial/ethnic (Hispanic 34.2%, Black 32.1%) and gender (women 81.1%) | Survey: Building Up a Diverse Workforce for Biomedical Research Trial (Building Up) with structured and open-ended questions | - Most participants experienced increased stress due to the COVID-19 pandemic. - Some differences were noted between underrepresented PhD researchers and physician-scientists. Physician-scientists reported that increased clinical demands, research delays, and the potential to expose family members to COVID-19 caused psychological distress, specifically. PhD researchers, more than physician-scientists, reported increased productivity (27% vs 9%), schedule flexibilities (49% vs 25%), and more quality time with friends and family (40% vs 24%) | There should be future evaluation of programs that address COVID-19 challenges among PhD researchers and physician scientists. Participant experienced increased stress, however no non-minority comparison group mentioned. |
| Dyrbye et al. (2007)^25^; US | Quantitative descriptive | Medical students (n=1689) | Racial/ethnic (24%) | Survey: MBI,^7^ the Primary Care Evaluation of Mental Disorders (PRIME MD),^26^ and the Medical Outcomes Study Short Form (SF-8)^27^ | - Non-minority students had greater depersonalization scores than minority students (p=0.01). - Minority and non-minority students scored similarly in terms of emotional exhaustion (p=0.1) and personal accomplishment (p=0.14). - Burnout adjusted for age, sex, parenting and marital status remained significantly greater in non-minority students (p<0.01). | Non-minority medical students were more likely to report burnout. |
| Dyrbye et al. (2006)^28^; US | Quantitative descriptive | Medical students (n=545) | Racial/ethnic (minority 16%) | Survey: MBI,^7^ PRIME MD,^26^ SF-8^27^ | - Symptoms of depression and burnout were similar among minority and non-minority students. - Minority students were more likely to report a low sense of personal accomplishment (p-0.02) and lower quality of life (p<0.05). | There was similar overall burnout between minority and non-minority medical students, however minority students reported a lower sense of personal accomplishment and quality of life. |
| Eliason et al. (2018)^29^; US | Mixed-methods | Healthcare professionals including physicians, physician associates, nurses, students/trainees mental health professionals, pharmacists, physical therapists, lab technicians, social workers, chaplains, and public health professionals (n=277) | Sexual (lesbian or gay 62%, bisexual 10%, heterosexual 10%, other 18%) and gender (women 48%, men 39%, transgender or gender nonconforming 13%) | Survey: quantitative (the Brief Resilience Scale^30^) and open-ended questions | - Most prevalent theme noted was discrimination. All respondents who identified as transgender faced discrimination. Some providers felt uneasy coming out in fear of making their patients or coworkers uncomfortable. - The second most prominent theme was worrying about the ramifications of being out at work such as changes in coworker and patient-provider relations. - In terms of coping, 47 participants mentioned educating others. Some unhealthy mechanisms were also mentioned, however not as commonly as health coping, such as ignoring comments and drinking alcohol. | Many gender and sexual minority healthcare professions fear being out in the work place and experience discrimination among other ramifications of their minority status. |
| Evans et al. (2021)^31^; US | Quantitative descriptive | Social work doctoral students (n=297) | Racial/ethnic (Black 14.1%)), gender (women 80.1%), sexual (30.3%) | Survey: CBI,^11^ Brief Symptom Inventory (BSI),^32^ Supportive People and Places Index^33^ | - Sexual minority respondents more frequently reported depressive symptoms (p<0.001) and anxiety symptoms (p<0.001) in comparison to heterosexual respondents. Heterosexual respondents reported higher levels of emotional connection to loved ones (p<0.001). - White students reported more depressive (p=0.01) and anxiety symptoms (p<0.001) compared to Black students. Black and Asian respondents reported higher levels of connection to loved ones as compared to white respondents (p<0.001). - No significant differences in depressive symptoms, anxiety symptoms, or connection to loved ones were noted by gender. | Sexual minority doctoral students reported worse well-being outcomes in comparison to heterosexual students. However, students of color reported lower levels of depressive and anxiety symptoms in comparison to white counterparts. Thus, well-being outcomes seem to vary by type of minority status. |
| Ey et al. (2013)^34^; US | Quantitative descriptive | Medical residents and fellows (n=450) | Racial/ethnic (non-white 24.5%) and gender (women 58%) | Intervention: Resident Wellness Program (RWP) that offered on-site free, unlimited counseling services  Survey: de novo questions regarding perceived barriers | - Logistic regression of perceived barriers to the program found that women were more concerned about taking a break to access counseling. Time remained the largest barrier. - More than 80% of respondents were more than “somewhat likely” to consider using the program. - Men (OR=0.54, 95% CI [0.34,0.84]) and racial/ethnic minority trainees (OR=0.49, 95% CI [0.28,0.85]) were more unwilling to seek help. | A wellness program for residents that offered free counseling services removed barriers of cost and knowledge about counseling services, but time remained a significant concern. Further evaluation is required to assess sex and ethnic differences in program utilization and stigma surrounding counseling. |
| Frias and Yuen (2021)^35^; US | Quantitative descriptive | Physician associate students (n=6,768) | Racial/ethnic (URM 21.2%) and gender (women 75.3%) | Survey: Perceived Stress Scale 4 (PSS-4)^36^ and an adapted version of the Psychological Sense of School Membership (PSSM) Scale^37^ | - URM students reported experiencing higher stress (p<0.001) and less PSSM (p=0.023) than their non-URM counterparts. - Men reported less stress than women (p=0.001). | Physician associate students who are URM and women report greater stress than their counterparts. |
| Garcia et al. (2020)^38^; US | Quantitative descriptive | Physicians (n=4,424) | Racial/ethnic (non-Hispanic white 78.7%, non-Hispanic Asian 12.3%, Hispanic/Latinx 6.3%, non-Hispanic Black 2.8%) | Survey: MBI,^7^ PRIME-MD,^26^ 5-point Likert-type scale survey items for work life balance and career satisfaction | - Black physicians were more likely to practice in primary care (p<0.001). - After adjusting for sex, age, clinical specialty, hours worked per week, primary practice setting, and relationship status, white physicians were more likely to experience burnout than physicians of minority racial/ethnic groups. The adjusted odds of burnout was 37% lower in Hispanic physicians and 51% lower in Black physicians in comparison to white physicians. | Physicians identifying as Hispanic/Latinx, Black, or Asian were less likely to report burnout when compared with white physicians. |
| Glymour et al. (2004)^39^; US | Quantitative descriptive | Physicians (n=2,217) | Racial/ethnic (Black 3%, Hispanic 6%, Asian or Pacific Islander 18%, white 73%) | Survey: Physicians’ Worklife Survey (PWS)^40,41^ | - Asian or Pacific Islander physicians reported lower job satisfaction (p<0.01) and higher stress (p<0.01) than white respondents. There was no significant difference in career satisfaction. - Hispanic physicians reported greater job (p=0.05) and career (p=0.03) satisfaction than white respondents. There was no significant difference in reported stress. - There were no significant differences between Black and white physicians in regards to job satisfaction, career satisfaction, or stress. | A national survey of physicians showed that job satisfaction differed by race/ethnicity where Hispanic physicians reported higher, Asian physicians reported lower, and Black physicians reported similar satisfaction compared to white physicians. |
| Graham-Brown et al. (2021)^42^; UK | Quantitative descriptive | Renal medicine higher specialty trainees (n=627) | Racial/ethnic (Black, Asian and minority ethnic (BAME) backgrounds) and gender | Survey: General Medical Council national training survey 2012-2019, CBI^11^ | - Men renal trainees reported higher burnout rates than women colleagues. - Burnout rates were higher for racial/ethnic minority or BAME trainees when compared to trainees identifying as white. | Racial/ethnic minority medical trainees reported higher burnout rates than white trainees. |
| Greenberg et al. (2022)^43^; US | Quantitative descriptive | Medical residents (n=300) | Racial/ethnic (residents of color (ROC) 41%, URiM 18%) and gender (non-men 40%) | Survey: Mental Health Continuum^44^ for flourishing, MBI,^7^ Cognitive and Affective Mindfulness Scale,^45^ Demand Control Support Questionnaire,^46^ PHQ,^3^ PSS,^36^ State-Trait Anxiety Index^47^ | - ROC have significantly greater anxiety (p=0.04) and lower flourishing (p=0.05) when compared to white residents. - UIM residents have lower depersonalization (p=0.02) and emotional exhaustion (p=0.05) than residents overrepresented in medicine (OIM). | Residents of color report lower global well-being than white residents. However, UIM residents report lower burnout than OIM residents. These somewhat conflicting findings support that evaluating “the specific contributors  and detractors of [well-being] among different individuals and groups is critical.”^43^ |
| Jaishankar et al. (2021)^48^; US | Quantitative non-randomized | Medical students (n=3,826) | Racial/ethnic (no reported percentages) | Survey: MBI,^7^ PSS,^36^ and three-question UCLA (University of California Los Angeles) Loneliness Scale^49^ | - Significant differences were found in burnout (p=0.004) and stress (p<0.0001) by race with Black, Asian, or other racial minority students reporting the highest levels. | Racial/ethnic minority medical students had significantly greater burnout and stress. |
| Keshet and Popper-Giveon (2016)^50^; Israel | Qualitative | Nurses (n=13) | Racial/ethnic (Arab nurses in Israel 100%) | Interview: Face-to-face semi- structured, in-depth interviews of participants through snow-ball sampling | - Perceived difficulties particular to the nurses’ ethnic minority background include "stereotypical and offensive attitudes on the part of patients and their families, and tense working relationships with Jewish colleagues."^50^ | Arab nurses, an ethnic minority group within a predominantly Jewish Israeli health system, reported poor working relationships and discrimination. |
| Khan et al. (2021)^51^; Canada | Quantitative descriptive | Internal medicine physicians (n=249 complete responses) | Racial/ethnic (31%) and gender/sexual (women 49%, LGBTQ 6%) | Survey: MBI^7^ | - No significant difference in burnout among men and women respondents. - Women were more likely to report emotional exhaustion (p=0.03) and low personal accomplishment (p=0.03) than men. - No gender differences in depersonalization. - Minority physicians were more likely to report low personal accomplishment in comparison to white respondents (p=0.001). | Women reported more emotional exhaustion and low personal accomplishment than men. Minority physicians were more likely to report low personal accomplishment. However, a composite MBI burnout score showed no difference by gender or race/ethnicity. |
| Klingler and Marckmann (2016)^52^; Germany | Qualitative | Physicians (n=20) | Migrant (foreign-born and foreign-trained, 100%) – various countries of origin: Romania, Poland, Russian Federation, Greece, Libya, Iran, Syria | Interview: semi-structured interviews with recruitment via snowballing focused on difficulties experiences and existing and desired support structures | - Participants noted some difficulties with setting-specific competencies such as understanding everyday language and important cultural or historical contexts. - Additionally, some reported difficulties in some clinical competencies such as different tools and treatments from their home country. - In regards to interpersonal relations, some felt discriminated as a foreigner or had family issues impairing their professional performance. | Migrant physicians in Germany might face different difficulties given their foreign status “that should be addressed on various levels to ensure physician [well-being], retention, and quality of care.”^52^ |
| Lawrence et al. (2022)^53^; US | Systematic Review | Medical students, residents/interns, physicians, and university faculty; 16 articles | Racial/ethnic (URiM) | Articles reporting burnout through various tools: MBI,^7^ CBI,^11^ modified Compassion Fatigue and Satisfaction Self-Test for Healpers,^54,55^ or single-item measures | - Inconsistent findings were seen with some studies finding that URiM experience lower rates of burnout, while others found no difference or greater burnout among those URiM. | The authors call for increased research to “further examine burnout by race/ethnicity . . . and contextualize the findings within the structural and systemic barriers specific” to minority populations.^53^ |
| Mitchell et al. (2022)^56^; US | Quantitative descriptive | State and local health department employees (N=18,952) | Racial/ethnic | Survey: 2017 Public Health Workforce Interests and Needs Survey (PH WINS)^57^ | - At higher supervisory support levels, job satisfaction among minorities was lower than their white counterparts. - Job satisfaction levels increased more with increasing pay satisfaction among minorities than white respondents. - Race has no moderating effect on the job satisfaction-turnover intentions relationship. | Focusing on job satisfaction through efforts such as pay equity and support structures may reduce turnover among minority public health personnel. |
| Nfonoyim et al. (2021)^58^; US | Qualitative | Pediatric emergency medicine physicians (n=18) | Racial/ethnic (URiM 100%, Black 72.2%, Hispanic 27.8%) and gender (women 83.3%) | Interview: semi-structured open-ended interviews | - Three main themes were identified: (1) challenges related to race, (2) support systems, and (3) suggestions to improve diversity and inclusion. - Participants often noted microaggressions from colleagues and patients. - Women URiM noted to be perceived as “angry” and “intimidating” when attempting to assert themselves. - Pressure to prove themselves as a result of negative experiences “produced stress and feelings of isolation.” - Resilience strategies included formal mentorship and peer and family support. | Participants mentioned that there is a need to increase diversity and promote inclusive work environments to improve the well-being of physicians URiM. |
| Nunez-Smith et al. (2009)^59^; US | Quantitative descriptive | Physicians (n=529) | Racial/ethnic (white 59%, Black 17%, Asian 15%, Hispanic 16%) | Survey: items based on prior surveys to assess well-being through experiences of discrimination, job turnover, intention to leave, and career satisfaction | - Racial/ethnic-based discrimination experiences were associated with increased turnover. - Among physicians who experienced discrimination versus those who did not, there was significantly less career satisfaction (45% vs 88%, p<0.01) and greater turnover intention (40% vs 10%, p<0.001). | Workplace discrimination, experienced by racial/ethnic minority physicians, is association with poor outcomes such as job turnover, career dissatisfaction, and contemplation of career change. |
| Obichi et al. (2023)^60^; US | Quantitative descriptive | Registered nurses, advanced nurse practitioners, physicians, pharmacists, nursing assistants, dentists, and respiratory therapists (n=74) | Racial/ethnic (self-identifying minority 100%, Black or African American 86%) | Survey: investigator-developed assessing challenges related to knowledge of rights, coping, stigma, support, self-advocacy, self-efficacy, and systemic drivers of poor mental health | - Some mental health challenges faced by MHCW included voicing their fears and maintaining household roles. - MHCW reported maintaining their mental health mostly through prayer sessions and family support time. - Study participants felt that they had some differential treatment than their white counterparts. | The authors propose that health systems need to dedicate resources to improve work conditions of MHCW and build an inclusive work environment. |
| Odei and Chino (2021)^61^; US | Quantitative descriptive | Radiation and medical oncologists (n=519) | Racial/ethnic and gender | Survey: 2017-2020 by the Association of American Medical Col- leges | - Minority radiation oncologists reported greater burnout rates than non-minority, however minority medical oncologists reported lower burnout rates than non-minority respondents. - Women radiation and medical oncologists were more burned out and less satisfied with their work than their respective men counterparts. | There is somewhat conflicting differences in burnout among racial/ethnic minority oncologists. Women uniformly reported more burnout. |
| Padela et al. (2016)^62^; US | Quantitative descriptive | Physicians (n=255) | Religious (self-identify with Islam) | Survey: various adapted items assessing religious discrimination, religious accommodation, and discrimination-related job turnover | - 45% of respondents rarely ever experienced discrimination and 24% reported experiencing it sometimes or more often. - 14% noted experiencing discrimination at their current workplace. - 47% reported that they perceive to be receiving greater scrutiny at work due to their religion. - Nearly three-fourths of respondents agreed that their workplace accommodates their religious identity. | This national survey provided some reassuring findings such as that most respondents’ workplaces are accommodating to their religion. However, some concerning findings include nearly half of respondents feeling as though they receive greater scrutiny due to their religion. |
| Perina et al. (2018)^63^; US | Quantitative descriptive | Emergency medicine resident physicians (n=766) | Racial/ethnic (non-White 20.9%) and gender (women 33.8%) | Survey: Longitudinal Study of Emergency Medicine Residents | - Minority and gender discrimination were among the least reported problems. | Emergency medicine physicians did not seem concerned with experiencing discrimination, however this sample is notably predominately comprised of white males. |
| Pillado et al. (2023)^64^; US | Quantitative descriptive | Vascular surgery trainees (n=510) | Racial/ethnic (white 53.1%, Asian 24.4%, Hispanic/Latinx 7.6%, Black 4.2%) |  | - Black and Asian respondents reported higher rates of racial/ethnic discrimination compared with the white, Hispanic/Latinx, and other/prefer not to say groups (p<0.001). - Upon multivariable analysis, female gender, Asian race, Black race, and training in the Southeastern United States were risk factors for discrimination. | Racial/ethnic discrimination is evident in vascular surgery training programs with Black and Asian trainees reporting higher rates than other groups. |
| Primack et al. (2010)^65^; US | Quantitative descriptive | Medical students, residents, fellows, predoctoral trainees, and faculty (n=179) | Racial/ethnic (white 65%, Asian 21%, URM 14%) and gender (women 50%) | Survey: de novo assessing burnout | - Burnout most common among URM (p=0.02). - Women reported higher burnout than men (p=0.03). | Higher burnout was reported among URM and women trainees. |
| Psenka et al. (2020)^66^; US | Quantitative descriptive | Family medicine residency program directors (n=268) | Racial/ethnic (white 84.8%, Asian 5.3%, Black 4.9%) and gender (women 48.1%) | Survey: 2018 survey conducted by the Council of Academic Family Medicine Educational Research Alliance (CERA) adapted questions from the Areas of Worklife Scale,^67^ the UCLA Loneliness scale,^49^ MBI,^7^ and PHQ-2^3^ | - No gender differences across distress measures. - On multivariable models, depressive symptoms were significant associated with race (Black, p<0.007). | The racial disparity is depressive symptoms calls for further analysis of potential contributors. |
| Rhead et al. (2020)^68^; UK | Quantitative descriptive | Healthcare practitioners: doctors, nurses, healthcare assistants, Improving Access to Psychological Therapy (IAPT) workers (n=931) | Racial/ethnic (Black 22%), gender (women 76%), migrant (37%), sexual (non-heterosexual 12%) | Survey: TIDES study^69^ utilized PHQ-9,^70^ GAD-7,^2^ PHQ-15,^71^ World Health Organization’s Health and Work Performance Questionnaire,^72^ Non-Illness Predictors of Sickness Absence Questionnaire^73^ | - Women were more likely to experience discrimination and harassment. - Black healthcare practitioners were more likely to experience and witness both discrimination and harassment. - No differences in discrimination and harassment reported by sexual orientation. | Discrimination and harassment is prevalent and affects well-being disproportionately more among women and Black racial groups. |
| Rivera (2018)^74^; US | Qualitative | LGBTQ+ mental health physicians (n=15) | Gender/sexual (LGBTQ+ 100%) | Interview: open-ended semi-structured | - Key work-related themes: social fear of being “othered”, lack of culturally diverse mentors, exposure to microaggressions, institutional ostracizing, creating a more diverse field | LGBTQ+ clinicians face unique stressors related to their minority identities that can act as barriers to professional practice. |
| Serrano et al. (2023)^75^; US | Quantitative descriptive | Student registered nurse anesthetists (SRNAs) | Ethnicity (Hispanic 13.6%), racial (white 60.7%, Black 15.0%), gender (women 71.4%) | Survey: de novo assessing well-being index measures | - There was a significant association between race/ethnicity and an increased incidence of discrimination (p<0.001) - SRNAs who have experienced at least one instance of discrimination had significantly higher well-being index scores than those who have never experienced discrimination (p<0.05). | Addressing discrimination critical to support wellbeing and retention of minority SRNAs. |
| Yoon et al. (2010)^76^; US | Quantitative descriptive | Obstetrician/gynecologist physicians (n=1154) | Race/ethnicity (Hispanic 6%, Black 6%, white 69&, Asian 18%), gender (women 47%), immigration history (born in the US 72%) | Survey: de novo questionnaire assessing conflict, MBI^7^ | - Female physicians were more likely to report physician-patient conflicts. - Foreign-born physicians were less likely to report such conflicts than US born respondents. - No association of conflict with race/ethnicity. | Conflict in a clinical encounter may represent burnout. Conflict is associated with immigration history and gender, but in opposite directions. |

Abbreviations: CI = confidence interval; HCW = healthcare worker; MHCW = minority healthcare worker; OR = odds ratio; UK = United Kingdom; US = United States

**References**

1. Abrahim H, Holman E. A scoping review of the literature addressing psychological well-being of racial and ethnic minority nurses during the COVID-19 pandemic. *Nurs Outlook*. 2023;71(1):101899. doi:10.1016/j.outlook.2022.11.003

2. Spitzer RL, Kroenke K, Williams JBW, Löwe B. A brief measure for assessing generalized anxiety disorder: The GAD-7. *Arch Intern Med*. 2006;166(10):1092-1097. doi:10.1001/archinte.166.10.1092

3. Kroenke K, Spitzer RL, Williams JBW. The Patient Health Questionnaire-2: validity of a two-item depression screener. *Med Care*. 2003;41(11):1284-1292. doi:10.1097/01.MLR.0000093487.78664.3C

4. Kovner C, Raveis VH, Van Devanter N, Yu G, Glassman K, Ridge LJ. The psychosocial impact on frontline nurses of caring for patients with COVID-19 during the first wave of the pandemic in New York City. *Nurs Outlook*. 2021;69(5):744-754. doi:10.1016/j.outlook.2021.03.019

5. Montoya V, Donnini K, Gauthier-Loiselle M, et al. Mental Health and Health-Related Quality of Life Among Nephrology Nurses: A Survey-Based Cross-Sectional Study. *NNJ*. 2021;48(5):447. doi:10.37526/1526-744X.2021.48.5.447

6. Alvandi M, Davis J. Risk Factors Associated with Burnout among Medical Faculty: A Systematic Review. *PIELEGNIARSTWO XXI WIEKU-NURSING IN THE 21 CENTURY*. Published online 2023. doi:10.2478/pielxxiw-2023-0030

7. (PDF) The Maslach Burnout Inventory Manual. Accessed April 9, 2024. https://www.researchgate.net/publication/277816643_The_Maslach_Burnout_Inventory_Manual

8. Adam S, Mohos A, Kalabay L, Torzsa P. Potential correlates of burnout among general practitioners and residents in Hungary: the significant role of gender, age, dependant care and experience. *BMC Family Practice*. 2018;19(1):193. doi:10.1186/s12875-018-0886-3

9. Hashmi AM. The challenge of Burnout in Public Medical Teachers in Pakistan: A mixed methods study. *Pak J Med Sci*. 2021;37(5):1268-1275. doi:10.12669/pjms.37.5.4429

10. Armstrong M, Reynolds K. Assessing Burnout and Associated Risk Factors in Medical Students. *J Natl Med Assoc*. 2020;112(6):597-601. doi:10.1016/j.jnma.2020.05.019

11. Kristensen TS, Borritz M, Villadsen E, Christensen KB. The Copenhagen Burnout Inventory: A new tool for the assessment of burnout. *Work & Stress*. 2005;19(3):192-207. doi:10.1080/02678370500297720

12. Bazargan-Hejazi S, Dehghan K, Chou S, et al. Hope, optimism, gratitude, and wellbeing among health professional minority college students. *Journal of American College Health*. 2023;71(4):1125-1133. doi:10.1080/07448481.2021.1922415

13. Boateng G, Schuster R, Boateng M. Uncovering a health and wellbeing gap among professional nurses: situated experiences of direct care nurses in two Canadian cities. *SOCIAL SCIENCE & MEDICINE*. 2019;242. doi:10.1016/j.socscimed.2019.112568

14. Burns KEA, Pattani R, Lorens E, Straus SE, Hawker GA. The impact of organizational culture on professional fulfillment and burnout in an academic department of medicine. *PLoS One*. 2021;16(6):e0252778. doi:10.1371/journal.pone.0252778

15. Trockel M, Bohman B, Lesure E, et al. A Brief Instrument to Assess Both Burnout and Professional Fulfillment in Physicians: Reliability and Validity, Including Correlation with Self-Reported Medical Errors, in a Sample of Resident and Practicing Physicians. *Acad Psychiatry*. 2018;42(1):11-24. doi:10.1007/s40596-017-0849-3

16. Carthon JMB, Travers JL, Hounshell D, Udoeyo I, Chittams J. Disparities in Nurse Job Dissatisfaction and Intent to Leave: Implications for Retaining a Diverse Workforce. *The Journal of nursing administration*. 2021;51(6):310-317. doi:10.1097/NNA.0000000000001019

17. Cedeño B, Shimkin G, Lawson A, Cheng B, Patterson DG, Keys T. Positive yet problematic: Lived experiences of racial and ethnic minority medical students during rural and urban underserved clinical rotations. *J Rural Health*. 2023;39(3):545-550. doi:10.1111/jrh.12745

18. Chilakala A, Camacho-Rivera M, Frye V. Experiences of race- and gender-based discrimination among Black female physicians. *Journal of the National Medical Association*. 2022;114(1):104-113. doi:10.1016/j.jnma.2021.12.008

19. Chin RW, Chua Y, Chu M, et al. Prevalence of Burnout among Universiti Sains Malaysia Medical Students. *Education in Medicine Journal*. 2016;8:61-74. doi:10.5959/eimj.v8i3.454

20. Daley S, Wingard DL, Reznik V. Improving the retention of underrepresented minority faculty in academic medicine. *J Natl Med Assoc*. 2006;98(9):1435-1440.

21. Doede M. Race as a predictor of job satisfaction and turnover in US nurses. *J Nurs Manag*. 2017;25(3):207-214. doi:10.1111/jonm.12460

22. Health Resources and Services Administration (HRSA). The Registered Nurse Population: Findings from the 2008 National Sample Survey of Registered Nurses. Published online 2010. https://data.hrsa.gov/DataDownload/NSSRN/GeneralPUF08/rnsurveyfinal.pdf

23. Douglas M, Coman E, Eden AR, Abiola S, Grumbach K. Lower Likelihood of Burnout Among Family Physicians From Underrepresented Racial-Ethnic Groups. *Ann Fam Med*. 2021;19(4):342-350. doi:10.1370/afm.2696

24. Doyle JM, Morone NE, Proulx CN, et al. The impact of the COVID-19 pandemic on underrepresented early-career PhD and physician scientists. *J Clin Transl Sci*. 2021;5(1):e174. doi:10.1017/cts.2021.851

25. Dyrbye LN, Thomas MR, Eacker A, et al. Race, Ethnicity, and Medical Student Well-being in the United States. *Archives of Internal Medicine*. 2007;167(19):2103-2109. doi:10.1001/archinte.167.19.2103

26. Utility of a New Procedure for Diagnosing Mental Disorders in Primary Care: The PRIME-MD 1000 Study | JAMA | JAMA Network. Accessed April 9, 2024. https://jamanetwork.com/journals/jama/article-abstract/383992

27. Ware JE, Kosinski M, Dewey JE, Gandek B. *How to Score and Interpret Single-Item Health Status Measures : A Manual for Users of the of the SF-8 Health Survey*. QualityMetric, Inc.; 2001.

28. Dyrbye LN, Thomas MR, Huschka MM, et al. A multicenter study of burnout, depression, and quality of life in minority and nonminority US medical students. *Mayo Clinic Proceedings*. 2006;81(11):1435-1442. doi:10.4065/81.11.1435

29. Eliason MJ, Streed CJ, Henne M. Coping With Stress as an LGBTQ+ Health Care Professional. *Journal of homosexuality*. 2018;65(5):561-578. doi:10.1080/00918369.2017.1328224

30. Smith BW, Dalen J, Wiggins K, Tooley E, Christopher P, Bernard J. The brief resilience scale: assessing the ability to bounce back. *Int J Behav Med*. 2008;15(3):194-200. doi:10.1080/10705500802222972

31. Evans KE, Holmes MR, Prince DM, Groza V. Social work doctoral student well-being during the COVID-19 Pandemic: A descriptive study. *International Journal of Doctoral Studies*. 2021;16:569-592. doi:10.28945/4840

32. Derogatis LR, Melisaratos N. The Brief Symptom Inventory: an introductory report. *Psychol Med*. 1983;13(3):595-605.

33. King JA. *Supportive People and Places Index*. Case Western Reserve University; Center on Trauma & Adversity

34. Ey S, Moffit M, Kinzie JM, Choi D, Girard DE. “If you build it, they will come”: attitudes of medical residents and fellows about seeking services in a resident wellness program. *J Grad Med Educ*. 2013;5(3):486-492. doi:10.4300/JGME-D-12-00048.1

35. Frias D, Yuen CX. The Physician Assistant Student Experience: Diversity, Stress, and School Membership. *J Physician Assist Educ*. 2021;32(2):113-115. doi:10.1097/JPA.0000000000000362

36. Cohen S, Kamarck T, Mermelstein R. A Global Measure of Perceived Stress. *Journal of Health and Social Behavior*. 1983;24(4):385-396. doi:10.2307/2136404

37. Goodenow C. The psychological sense of school membership among adolescents: Scale development and educational correlates. *Psychology in the Schools*. 1993;30(1):79-90. doi:10.1002/1520-6807(199301)30:1<79::AID-PITS2310300113>3.0.CO;2-X

38. Garcia LC, Shanafelt TD, West CP, et al. Burnout, depression, career satisfaction, and work-life integration by physician race/ethnicity. *JAMA Network Open*. 2020;3(8):e2012762. doi:10.1001/jamanetworkopen.2020.12762

39. Glymour MM, Saha S, Bigby J. Physician race and ethnicity, professional satisfaction, and work-related stress: results from the Physician Worklife Study. *J Natl Med Assoc*. 2004;96(10):1283-1289, 1294.

40. Konrad TR, Williams ES, Linzer M, et al. Measuring physician job satisfaction in a changing workplace and a challenging environment. SGIM Career Satisfaction Study Group. Society of General Internal Medicine. *Med Care*. 1999;37(11):1174-1182. doi:10.1097/00005650-199911000-00010

41. Williams ES, Konrad TR, Linzer M, et al. Refining the Measurement of Physician Job Satisfaction: Results From the Physician Worklife Survey. *Medical Care*. 1999;37(11):1140.

42. Graham-Brown MP, Beckwith HK, O’Hare S, Trewartha D, Burns A, Carr S. Impact of changing medical workforce demographics in renal medicine over 7 years: Analysis of GMC national trainee survey data. *Clin Med (Lond)*. 2021;21(4):e363-e370. doi:10.7861/clinmed.2020-1065

43. Greenberg AL, Cevallos JR, Ojute FM, Davis DL, Greene WR, Lebares CC. The General Surgery Residency Experience: A Multicenter Study of Differences in Wellbeing by Race/Ethnicity. *Ann Surg Open*. 2022;3(3):e187. doi:10.1097/AS9.0000000000000187

44. Corey L. M. Keyes. The Mental Health Continuum: From Languishing to Flourishing in Life. *Journal of Health and Social Behavior*. 2002;43(2):207-222. doi:10.2307/3090197

45. Mindfulness and Emotion Regulation: The Development and Initial Validation of the Cognitive and Affective Mindfulness Scale-Revised (CAMS-R) | Journal of Psychopathology and Behavioral Assessment. Accessed April 11, 2024. https://link.springer.com/article/10.1007/s10862-006-9035-8

46. Sanne B, Torp S, Mykletun A, Dahl AA. The Swedish Demand-Control-Support Questionnaire (DCSQ): factor structure, item analyses, and internal consistency in a large population. *Scand J Public Health*. 2005;33(3):166-174. doi:10.1080/14034940410019217

47. Spielberger C, Gorsuch R, Lushene R. *Manual for the State-Trait Anxiety Inventory*. Consulting Psychologists Press; 1970.

48. Jaishankar D, Dave S, Tatineni S, et al. Burnout, stress, and loneliness among u.s. medical students during the COVID-19 pandemic: A national survey. *Journal of General Internal Medicine*. 2021;36(SUPPL 1):S161. doi:10.1007/s11606-021-06830-5

49. Hughes ME, Waite LJ, Hawkley LC, Cacioppo JT. A Short Scale for Measuring Loneliness in Large Surveys. *Res Aging*. 2004;26(6):655-672. doi:10.1177/0164027504268574

50. Keshet Y, Popper-Giveon A. Work experiences of ethnic minority nurses: a qualitative study. *ISRAEL JOURNAL OF HEALTH POLICY RESEARCH*. 2016;5. doi:10.1186/s13584-016-0076-5

51. Khan N, Palepu A, Dodek P, et al. Cross-sectional survey on physician burnout during the COVID-19 pandemic in Vancouver, Canada: the role of gender, ethnicity and sexual orientation. *BMJ OPEN*. 2021;11(5). doi:10.1136/bmjopen-2021-050380

52. Klingler C, Marckmann G. Difficulties experienced by migrant physicians working in German hospitals: a qualitative interview study. *Human Resources for Health*. 2016;14:57-57.

53. Lawrence J, Davis B, Corbette T, Hill E, Williams D, Reede J. Racial/Ethnic Differences in Burnout: a Systematic Review. *JOURNAL OF RACIAL AND ETHNIC HEALTH DISPARITIES*. 2022;9(1):257-269. doi:10.1007/s40615-020-00950-0

54. Stamm BH. *Measuring Compassion Satisfaction as Well as Fatigue: Developmental History of the Compassion Fatigue and Satisfaction Test New York: Brunner*. Routledge; 2002.

55. Figley CR. Compassion fatigue as secondary traumatic stress disorder: an overview, w: compassion fatigue, red. *New York: CR Figley*. Published online 1995.

56. Mitchell AK, Apenteng BA, Boakye KG. Examining Factors Associated With Minority Turnover Intention in State and Local Public Health Organizations: The Moderating Role of Race in the Relationship Among Supervisory Support, Job Satisfaction, and Turnover Intention. *J Public Health Manag Pract*. 2022;28(5):E768-E777. doi:10.1097/PHH.0000000000001571

57. Public Health Workforce Interests and Needs Survey: 2017 Findings. de Beaumont Foundation and Association of State and Territorial Health Officials (ASTHO). Accessed April 12, 2024. https://debeaumont.org/phwins/findings/

58. Nfonoyim B, Martin A, Ellison A, Wright JL, Johnson TJ. Experiences of underrepresented faculty in pediatric emergency medicine. *Acad Emerg Med*. 2021;28(9):982-992. doi:10.1111/acem.14191

59. Nunez-Smith M, Pilgrim N, Wynia M, et al. Health Care Workplace Discrimination and Physician Turnover. *JOURNAL OF THE NATIONAL MEDICAL ASSOCIATION*. 2009;101(12):1274-1282. doi:10.1016/S0027-9684(15)31139-1

60. Obichi CC, Omenka O, Perkins SM, Oruche UM. Experiences of Minority Frontline Healthcare Workers During the COVID-19 Pandemic. *J Racial Ethn Health Disparities*. Published online 2023. doi:10.1007/s40615-023-01833-w

61. Odei B, Chino F. Incidence of Burnout Among Female and Minority Faculty in Radiation Oncology and Medical Oncology. *International Journal of Radiation Oncology, Biology, Physics*. 2021;111(3):S28-S29. doi:10.1016/j.ijrobp.2021.07.092

62. Padela AI, Adam H, Ahmad M, Hosseinian Z, Curlin F. Religious identity and workplace discrimination: A national survey of American Muslim physicians. *AJOB Empirical Bioethics*. 2016;7(3):149-159. doi:10.1080/23294515.2015.1111271

63. Perina DG, Marco CA, Smith-Coggins R, Kowalenko T, Johnston MM, Harvey A. Well-Being among Emergency Medicine Resident Physicians: Results from the ABEM Longitudinal Study of Emergency Medicine Residents. *The Journal of emergency medicine*. 2018;55(1):101-109.e2. doi:10.1016/j.jemermed.2018.04.003

64. Pillado E, Li R, Eng J, et al. Persistent racial discrimination among vascular surgery trainees threatens wellness. *JOURNAL OF VASCULAR SURGERY*. 2023;77(1):262-268. doi:10.1016/j.jvs.2022.09.011

65. Primack BA, Dilmore TC, Switzer GE, et al. Burnout among early career clinical investigators. *Clinical and Translational Science*. 2010;3(4):186-188. doi:10.1111/j.1752-8062.2010.00202.x

66. Psenka TM, Freedy JR, Mims LD, et al. A cross-sectional study of United States family medicine residency programme director burnout: implications for mitigation efforts and future research. *Fam Pract*. 2020;37(6):772-778. doi:10.1093/fampra/cmaa075

67. Leiter M, Maslach C. Areas of Worklife: A Structured Approach to Organizational Predictors of Job Burnout. In: *Research in Occupational Stress and Well-Being*. Vol 3. ; 2004:91-134. doi:10.1016/S1479-3555(03)03003-8

68. Rhead RD, Chui Z, Bakolis I, et al. Impact of workplace discrimination and harassment among National Health Service staff working in London trusts: Results from the TIDES study. *BJPsych Open*. 2020;7(1). doi:10.1192/bjo.2020.137

69. TIDES Study – Tackling inequalities and discrimination experiences in health services. Accessed April 12, 2024. https://tidesstudy.com/

70. Kroenke K, Spitzer RL, Williams JBW. The PHQ-9. *J Gen Intern Med*. 2001;16(9):606-613. doi:10.1046/j.1525-1497.2001.016009606.x

71. Kroenke K, Spitzer RL, Williams JBW. The PHQ-15: validity of a new measure for evaluating the severity of somatic symptoms. *Psychosom Med*. 2002;64(2):258-266. doi:10.1097/00006842-200203000-00008

72. Kessler RC, Barber C, Beck A, et al. The World Health Organization Health and Work Performance Questionnaire (HPQ). *J Occup Environ Med*. 2003;45(2):156-174. doi:10.1097/01.jom.0000052967.43131.51

73. Harvey SB, Wang MJ, Dorrington S, et al. NIPSA: a new scale for measuring non-illness predictors of sickness absence. *Occup Environ Med*. 2018;75(2):98-104. doi:10.1136/oemed-2017-104382

74. Rivera AD. *Perceived Stigma and Discrimination as Barriers to Practice for LGBTQ Mental Health Clinicians: A Minority Stress Perspective*. ProQuest Information & Learning; 2018. https://search.ebscohost.com/login.aspx?direct=true&AuthType=ip,shib&db=psyh&AN=2017-43828-114&site=ehost-live&scope=site&authtype=ip,shib&custid=s3555202

75. Serrano Y, Dalley CB, Crowell NA, Eshkevari L. Racial And Ethnic Discrimination During Clinical Education and Its Impact on the Well-Being of Nurse Anesthesia Students. *AANA J*. 2023;91(4):259-266.

76. Yoon J, Rasinski K, Curlin F. Conflict and emotional exhaustion in obstetrician-gynaecologists: a national survey. *JOURNAL OF MEDICAL ETHICS*. 2010;36(12):731-735. doi:10.1136/jme.2010.037762
